# A Cross-Sectional Review of HIV Screening in High-Acuity Emergency Department Patients: A Missed Opportunity

**DOI:** 10.5811/westjem.18067

**Published:** 2024-08-01

**Authors:** Jacqueline J. Mahal, Fernando Gonzalez, Deirdre Kokasko, Ahava Muskat

**Affiliations:** * Jacobi Medical Center, Department of Emergency Medicine, Bronx, New York; † Albert Einstein College of Medicine, Bronx, New York

## Abstract

**Introduction:**

Emergency department (ED) patients requiring immediate treatment often bypass a triage process that includes HIV screening. In this study we aimed to investigate the potential missed opportunity to screen these patients for HIV.

**Methods:**

We conducted this cross-sectional study in a municipal ED over a six-week period between June–August 2019. The patient population in this study arrived in the ED as a pre-notification from prehospital services or designated by the ambulance or walk-in triage nurse as requiring immediate medical attention. Medical student researchers collected demographic data and categorized patients into three clinical groups (trauma, medical, psychiatric). They documented the patient’s eligibility for HIV screening as determined by a physician and confirmed that the patient met criteria of clear mental status, controlled pain, stable vital signs, and ability to contribute to a medical history and physical examination. The student researchers did this at initial presentation and then again during the patient’s ED stay of up to eight hours. The study outcomes measured the percentage of total patients within each clinical group (trauma, medical, psychiatric) able to engage in the HIV screening process upon arrival and during an eight-hour ED stay.

**Results:**

On average, 700 patients per month are announced on arrival via overhead page, indicating that they require immediate medical attention. During the six-week study, 205 patients (approximately 20% of total) were enrolled: 114 trauma; 56 medical; and 35 psychiatric presentations. The average patient age was 53; 60% of patients were male. Niney-eight (48%) patients were eligible for HIV screening within an eight-hour ED stay; 63 (31%) were able to be screened upon initial presentation and 35 (17%) in the first eight hours of their ED visit. Within medical and trauma subgroups, there was no significant difference in the proportion (36%) of patients that could be screened upon presentation. Among the psychiatric presentations, only five (14%) were able to be screened during their hospital stay.

**Conclusion:**

Triage protocols for high-acuity medico-surgical patients resulted in a missed opportunity to screen 48% of patients for HIV. Acute psychiatric patients represented a particular missed opportunity. We advocate for universal HIV screening, facilitated through electronic best practice advisories and a modified triage tailored to higher acuity patients. Implementing these changes would ensure that HIV screening is not overlooked in high-acuity ED patients, leading to early detection and timely interventions.

Population Health Research Capsule What do we already know about this issue?

*While universal HIV screening in the ED is a well-known and reviewed clinical activity, acutely injured and medically ill patients are often excluded.*

What was the research question?

*Do patients who present emergently to an urban ED present a missed opportunity for HIV screening?*

What was the major finding of the study?

*Of the 205 acutely ill, injured or psychiatric patients in this study who bypassed typical HIV screening in triage, 98 (48%) were screened for HIV during their eight-hour ED stay, with 63 (31%) screened upon initial presentation.*

How does this improve population health?

*This study highlights a gap in HIV screening and a missed opportunity for testing HIV in ED patients.*

## INTRODUCTION

The US Centers for Disease Control and Prevention (CDC) reported 131.3 million visits to United States emergency departments (ED) in 2020.^1^ In 2014, 7% of patients who visited the ED reported a lack of access to clinicians rather than seriousness of their medical condition as the reason for their last ED visit.^2^ Approximately 1.2 million people live with HIV in the US, and 13% are unaware of the diagnosis.^3^ This incidence of HIV infections, coupled with significant ED volume and use of the ED for primary care, continues to make the ED a critical point of engagement with the medical system and, thus, an opportunity to provide HIV screening.^4^ ^–^ ^7^

Since the CDC’s 2006 recommendation for opt-out HIV screening for patients in all healthcare settings, there has been ample literature on universal HIV screening in the ED; however, acutely ill patients are often excluded from data collection.^8^ ^–^ ^10^ One study focusing on HIV screening of acutely ill medical patients in the ED found that the majority of the patients diagnosed with HIV were admitted with AIDS and had an average of three previous healthcare visits prior to HIV screening.^11^ When considering trauma patients in the ED, the literature reports HIV screening rates that range from 25.2–64.1%.^12^ ^–^ ^14^ A recent paper comparing screening in trauma to medicine patients found that screening in trauma patients was lower than in medical patients, yet HIV rates were higher in trauma vs medical patients.^14^ Both studies demonstrate that it is feasible to test these higher acuity patients and suggest that high-acuity patients may be another missed opportunity in the ED to identify previously undiagnosed HIV.

In our setting, if a patient is acutely ill or injured requiring immediate medical attention, the patient is announced via an overhead intercom and is moved to a resuscitation bay, bypassing the triage process that includes required HIV screening. Per New York State Public Health Law (PHL),^15^ there are three exceptions to the required HIV testing offer: life-threatening illness; recent testing and no recent risk behaviors; and a determination by the attending that the patient does not have mental capacity. We hypothesize that our triage process of automatically excluding patients identified via overhead page presents missed opportunities to screen otherwise eligible patients.

## METHODS

### Design

This was a single-site, cross-sectional study. Our objective was to measure what percentage of patients deemed acute, and who thus bypassed the triage process that includes HIV screening in order to receive immediate medical evaluation, were able to be screened for HIV during their ED stay. The protocol was approved by the Albert Einstein College of Medicine and the New York City Health & Hospitals institutional review boards and was deemed exempt from requiring consent. This study is reported using the Strengthening the Reporting of Observational Studies in Epidemiology (STROBE) guidelines.^16^

### Setting

This study was conducted in a municipal, adult ED with Level 1 trauma designation in New York City with an annual census of approximately 73,000 and approximately 15% of patients arriving by ambulance. Enrollment occurred over a six-week period from June–August 2019. Three medical student researchers (SR) were present in the ED for approximately 13 hours per day, 5–7 days per week.

### Research Workflow

The patient population in this study arrived in the ED as a pre-notification from prehospital services or was designated by the ambulance or walk-in triage nurse as requiring immediate medical attention. These patients were either Emergency Severity Index (ESI) 1 or 2, were announced via an overhead intercom system, and moved to a resuscitation bay. In real time, SRs reported to the resuscitation bay to record demographic data including age, gender, chief complaint, date, and time of presentation. They assigned patients to one of three clinical groups: trauma; medical; or psychiatric. Psychiatric patients included those with agitation secondary to substance use, primary psychiatric presentation, or a dual diagnosis. The attending or resident physician determined whether the patient could engage in the HIV screening process, and the SRs checked that the patient met criteria of clear mental status, controlled pain, stable vital signs, and ability to contribute to medical history and physical examination.

If a patient could not engage in the HIV screening process, but an appropriate healthcare proxy (HCP) was present to provide consent, the patient was considered screenable. Verbal consent of the patient or the patient proxy is required prior to ordering the HIV test in accordance with New York State PHL 2781/2781a^17^; however, asking the HCP for consent for HIV testing is not typically done in our ED. Patients who were not immediately able to participate in the HIV screening process were reassessed at four and eight hours after presentation. Eight hours was chosen since the average length of stay in this ED is approximately seven hours.

We were unable to receive HIV test results for patients who were both eligible and opted in for testing. The study period straddled a hospital-wide transition to a new electronic health record system (EHR), which included a change in the HIV testing protocol, leading to lost and canceled blood tests.

### Data Analysis

The primary study outcomes were the percentages of patients in each of the clinical groups (trauma, medical, psychiatric) who were able to engage in the HIV test screening process at arrival and by or before eight hours. Mean age with standard deviation were reported for each clinical group and compared to the mean of the entire cohort. Since consent via HCP is atypical in practice, we report the number of patients and the average age of this patient subgroup.

We compared the proportion of patients in each clinical group and the proportion that could engage in the HIV screening at arrival and by or before eight hours using the trauma group as the reference group. We used χ^2^ to compare proportions and the Student *t*-test to compare means with the α at .05 or less for two-tailed tests of significance. Analysis was completed in Excel 2019 (Microsoft Corporation, Redmond, WA).

## RESULTS

In our ED, approximately 700 patients per month are called overhead on arrival and moved to the resuscitation bay. During the six-week study period, 205 patients were enrolled, capturing approximately 20% of overhead notifications. The average time of day the SR responded to the overhead page was 3:40 pm with 23% of patients being seen after 8 pm on the overnight shift. A mean male age of 44.7 vs a mean female age of 66.6 was statistically significant in the trauma group only (*P* < .001, Table 1). The medical and psychiatric clinical subgroups had no statistically significant difference in age by gender. The proportion of patients in the medical clinical subgroup did not differ statistically from the trauma reference group (Table 1). Eight (4%) patients were included as screenable because a HCP provided consent; average age of these eight patients was 65 with a range of 23–91.

**Table 1.** Patient characteristics: age, gender, clinical presentation assignment.

	Number (N)	%	*P-*value^1^	Mean age	SD (±)			
Total patients	205			53	21			
Clinical subgroup				Mean age (male)	SD (±)	Mean age (female)	SD (±)	*P-*value^2^
Trauma	114	56%		44.7	19	66.6	21	<0.001
Medical	56	27%	0.78	55.3	19	64.8	20	0.09
Psychiatric	35	17%	<0.001	41.4	12	46.8	17	0.38

1 Medical and psychiatric clinical subtype were compared to the trauma group as a reference.

2 Mean age by gender were compared within each clinical sub-type.

Of the 205 patients, 98 (48%) were able to engage in HIV screening during their eight-hour ED stay. Of these 98 patients, 63 (31%) were able to be screened upon initial presentation and an additional 35 (17%) in the first eight hours of their ED visit. When categorized by presentation type, 61 (54%) of 114 trauma patients, 32 (57%) of 56 medical patients, and five (14%) of 35 psychiatric patients were able to engage in HIV screening during their eight-hour ED stay. There was no statistical difference between ability to participate in screening between trauma and medical clinical presentations (Table 2). Compared to trauma and medical patients, psychiatric notifications had a significantly lower ability to be screened by the eight-hour mark (*P* < .03). The patient’s level of psychiatric acuity, being in police custody, or leaving upon sobriety were reasons that 30 (86%) of the 35 psychiatric patients were not able to be screened within eight hours in the ED.

**Table 2.** Percentage of patients by presentation type and time who were able to be screened for HIV.

Presentation sub-type	Patients (total N = 205)	Screened at presentation (t = 0 hours)	*P-*value	Screened ≤ 8 hours	*P-*value
Trauma	114	41 (36%)		20 (18%)	
Medical	56	20 (36%)	0.97	12 (21%)	0.52
Psychiatric	35	2 (6%)	<0.001	3 (8%)	0.03

## DISCUSSION

This single-site, cross-sectional study demonstrated that 36% of patients who presented with emergent medical or trauma clinical presentations, thus bypassing HIV screening in triage, were able to be screened at initial presentation. An additional 21% who presented for medical and 18% for trauma presentations were able to be screened by eight hours into their ED stay. The results were statistically consistent between patients in the medical or trauma clinical presentation groups and statistically less likely for psychiatric patients. Notably, 86% of psychiatric patients were unable to be screened within eight hours in the ED.

With 1 in 7 people, or nearly 165,000 in the United States,^18^ unaware of their HIV status, universal, non-targeted HIV screening in high-volume settings like EDs remains an effective strategy. Studies from both Oakland and Chicago report that approximately 50% of new HIV diagnoses would have been missed had they used a targeted, symptom- and risk-based screening methodology.^19^ ^–^ ^23^ In a randomized clinical study comparing a targeted vs a non-targeted screening approach, Haukoos et al concluded that targeted screening was not superior, although it was more efficient with fewer tests completed.^24^

In addition to the screening methodology, the location of HIV screening may influence the completion of testing. Screening for HIV can occur during triage, registration, in the waiting room with kiosks and dedicated staff, or at the bedside driven by a clinician. In Tan et al, the authors reviewed 20 HIV testing protocols.^25^ They found that offer rates are highest during registration and at triage, attributed to systematic questioning, reaching 100% in some studies. The offer of testing does not, however, equate to acceptance of testing. The highest acceptance rates were found at the bedside and in the waiting room, often because the person doing the screening would also be conducting the test.^25^ Screening and testing protocols differ by site and resources available, making generalizability across all ED settings difficult.

Triage is one of the most important processes in the ED. To guide clinicians with this task, triage scores are used to provide an objective measure of patient acuity to focus on the sickest patients. The Emergency Severity Index (ESI) triage score is the most used in the US^26^ and is the one used in our institution. Studies report great variability with poor to moderate accuracy,^27^ ^,^ ^28^ especially in high-acuity patients. The American College of Surgeons Committee on Trauma recommends an over-triage rate of 25–50% on activation of trauma teams at trauma centers, and the literature reports a range of 18–91%.^29^ ^–^ ^32^ This variability may be necessary to ensure prompt treatment of life-threatening injuries and illnesses, while also reducing the number of acute patients treated at non-trauma centers.^29^

Over-triage and an emphasis on immediate intervention presumes ineligibility for HIV screening in our triage process that would typically include universal, non-targeted HIV screening. Patients with high acuity (ESI 1 and 2) were not informed of HIV screening in our study, as was the case in other studies.^15^ ^,^ ^33^ The focus on identifying, stabilizing, and treating acute injury or illness sensibly supersedes the HIV screening process. A true universal HIV screening protocol should include all patients regardless of ESI and include an individual assessment to determine ability to consent, rather than presumed ineligibility. While we had the manpower to reassess patients periodically, this resource-heavy model is not likely to be broadly replicable.

Even with the ability to reassess periodically, we found that 86% of acute psychiatric patients were not able to be screened for HIV at initial presentation or within eight hours in the ED. In all stages of the HIV care cascade, the patient population that struggles with mental health is met with challenges. Mental health disorders increase the risk of HIV acquisition by 4–10 times^34^ ^,^ ^35^ and, at the same time, interfere with HIV testing and learning one’s HIV status.^36^ The struggle with depression, anxiety, trauma, and substance use is a substantial barrier to HIV prevention methods (ie, condoms, pre-exposure prophylaxis), and adults with mental health disorders were more likely to be involved in behaviors associated with HIV acquisition or transmission than adults without mental disorders.^37^ ^,^ ^38^ Struggles with mental health contribute to poor retention in care and anti-retroviral adherence.^38^ ^,^ ^39^ Without the benefit of viral suppression achieved with anti-retroviral treatment, acute-care hospitalizations for patients with HIV and mental health disorders are higher than for HIV patients without mental health disorders.^39^ ^,^ ^40^ Of the acute patients presenting to the ED, our findings suggest that patients with mental health presentations and, in particular, acute mental health crises may require an alternative or additional approach to HIV screening and testing.

The ED is one place to start the care cascade with universal testing. However, we see that the acute patient, and especially the patient with acute mental health presentations, may require an alternative approach, other than during initial triage, to ensure that screening occurs. Using the EHR has been shown to optimize screening and testing and to increase identification of new HIV infections.^41^ ^,^ ^42^ Building an ESI 1 and 2 order set that includes an HIV test could be one means to address HIV screening into the care of acutely ill patients. The HIV test in the order set would require the clinician to acknowledge screening eligibility and verbal consent prior to finalizing the order. A best practice advisory with scripts for clinicians to use opt-out language could be programmed to fire if patients haven’t yet been screened during the current ED encounter and haven’t received HIV testing in a predetermined look-back period. This strategy could address any patient who may have missed HIV screening or testing in the ED, not just our acute patients who miss our triage screening. Any new approach or combination of approaches would require implementation plans and processes and future investigation before being accepted as solutions.

## LIMITATIONS

This was not a complete sample of all acute patients presenting to the ED during the study period since we did not have SRs 24 hours a day. This was a single-site study in a Level I trauma center with high volume, which contributes to problems with generalizability mainly for non-urban hospital settings. The approach to HIV screening is likely to be variable in other EDs and may not occur as part of the triage process. Differences in laws and regulations for testing and consent may also contribute to the lack of generalizability of these findings. The determination of being able to be screened is clinician-dependent, with possible bias toward HIV screening and testing in the ED and whether people should be asked. We are not able to report the disease prevalence in this small dataset. The HIV test results were not reported due to inconsistent testing and lab protocols for HIV testing as the hospital migrated to a new EHR system during the testing period. While not the focus of the study, we acknowledge that this data and collection of HIV risk factors for patients would have been a valuable addition to the study.

## CONCLUSION

This study highlights a gap in HIV screening in EDs and a missed opportunity for testing for HIV in ED patients. We found that close to 48% of patients who present for emergent care and missed the HIV universal screening that occurs during triage in our institution could engage in the screening process either at presentation or during their ED stay. And we identified an already vulnerable group—psychiatric patients— that appears to be ineligible for screening within an ED stay, leaving us to consider whether these patients will determine their HIV status. Future research is needed to assess the effectiveness of electronic best practice advisories and built-in HIV screening and testing order sets in higher acuity patients, as well as approaches to meeting the needs of the acute and vulnerable psychiatric patient.
